# Can access to urban networks promote urban development? Evidence from the Yangtze River Delta region of China

**DOI:** 10.1371/journal.pone.0300199

**Published:** 2024-03-12

**Authors:** Liang Ding, Zhiqiong Yang, Junshen Zhang, Yahui Chen, Xiaohan Wang

**Affiliations:** 1 School of Design and Architecture, Zhejiang University of Technology, Hangzhou, China; 2 China Academy of Housing and Real Estate, Zhejiang University of Technology, Hangzhou, China; 3 ZJU Qizhen Future City Tec (Hangzhou) Co., Ltd, Hangzhou, China; 4 College of Civil Engineering and Architecture, Zhejiang University, Hangzhou, China; 5 Zhejiang University Urban— Planning & Design Institute Co. Ltd, Hangzhou, China; East China Normal University, CHINA

## Abstract

The regional networking strategy is widely implemented in China as a normative policy aimed at fostering cohesion and enhancing competitiveness. However, the empirical basis for this strategy remains relatively weak due to limitations in measurement methods and data availability. This paper establishes the urban networks by the enterprise investment data, and then accurately measures the network’s external effects of each city by the method of MGWR model. The results show that: (1) Regional networking plays a significant role in urban development, although it is not the dominant factor. (2) The benefits of network connections may vary depending on the location and level of cities. (3) The major cities assume a pivotal role in the urban network. Based upon the aforementioned research conclusions, this paper presents strategic measures to enhance the network’s external impacts, aiming to offer insights for other regions in formulating regional development strategies and establishing regional urban networks.

## 1 Introduction

Under the background of informationization, globalization and regional integration, the cross-regional flow of production factors has become more frequent. Cities are increasingly interconnected with surrounding cities through information networks and rapid transportation routes [1, 2]. In light of this trend, many countries and regions actively promote strategies for regional networks development, including Belgium (’Urban Networks’), Denmark (’National Centres’), Switzerland (’Vernetzte Staidtes system’) and other multi-center strategies [3], while China implements various coordinated development strategies such as Beijing-Tianjin-Hebei Cooperated Development, Guangdong-Hong Kong-Macao Greater Bay Area construction, and Yangtze River Delta (YRD) integrated development. The primary objective is to establish an expanded spatial scope for regional cooperation, thereby facilitating the synchronized development of urban areas through enhanced and expedited flow of resources, ultimately narrowing the urban disparity. The efficacy of these strategies is pivotal in shaping future strategic decisions at the regional and national levels. Currently, there is a growing scholarly interest in empirically evaluating these strategic policies and investigating the potential of regional networking to facilitate coordinated urban development. They found that small and medium-sized cities could potentially internalize the benefits of larger cities by being well-positioned in urban networks [4, 5]. However, it should be noted that network connection can also have negative effects on the performance of cities due to competition processes, and not every city profits equally from high levels of network embeddedness [6]. A research paradigm for network externalities theory has been established through these studies, contributing to the advancement of academic discourse in this field. Nevertheless, due to the difficulty of data acquisition and the limitations of measurement methods, relevant research focuses on the measurement of network externalities in the whole region, while the investigation into the spatial heterogeneity of network externalities (i.e., the disparities in the external impacts derived from the network among cities of diverse locations and scales) has not been sufficiently discussed. Therefore, based on the theory of urban network externalities, this paper employs a Multi-scale Geographically Weighted Regression (MGWR) model to measure the network externalities of various cities in the YRD region and further reveals their spatial heterogeneity characteristics. By exploring the impact of regional network strategies on the development of various cities, it is helpful to improve the theoretical framework of China’s urban network, enrich empirical research on international urban network theory, and provide experience for the regional development of other developing countries.

## 2 Literature review

### 2.1 The theory of network externalities

Urban network externalities are employed to elucidate the impact of urban networks on regional urban development. The term "urban networks" refers to the intricate and diversified spatial structure resembling a network between cities, which is shaped by globalization and informatization. The theory of urban networks originates from the study of the global economic spatial organization known as the "world urban network". Part of the relevant research is grounded in the Actor Network Theory (ANT) [7–11], which conceptualizes cities as intricate networks comprising diverse actors, with enterprises being identified as the most significant actor. These studies employ various methodologies such as interviews and anthropological field surveys to conduct comprehensive investigations on senior managers of enterprises, unveiling the formation and evolution processes of enterprise networks and urban networks. The other part draws upon Social Networks Analysis (SNA) [12–14], which aims to quantify the urban network’s structure and unveil the underlying implications and connections more accurately. However, these studies primarily focus on describing network characteristics, and there remains a relative dearth of research examining the impact of networks on regional economic development. Camagni [15] and Capello [16] introduced the concept of "urban network externalities" into the field of urban network research, thereby expanding the theoretical exploration of the impacts of urban networks. The external effects associated with urban networks have progressively emerged as a central focus within this realm of study.

The theory of network externalities originates from economic research, referring to the additional benefits that accrue to each user as the number of users consuming a product or service changes in real economic activities. Camagni defined this externality beyond spatial distance as "urban network externality" from the perspective of enterprise spatial behavior decision-making, formally introduced the network externality into the research field of urban network. He emphasizes that urban network externality functions as a club product, benefiting only cities or entities connected to the network; Capello further expounded on Camagni’s concept from a macro perspective, emphasizing the complementary and synergistic connections between cities. He highlighted that network externalities underscore the impacts resulting from cities integrated into networks, achieving complementary, integrated, or synergistic effects through interconnecting nodes [17–19], encompassing both positive and negative aspects [16]. Scale borrowing is a prevalent positive externality observed in networks [20]. This concept was initially introduced Alonso, small cities situated within larger "metropolitan complexes" can enhance their development by harnessing the agglomeration benefits offered by neighboring large cities [21, 22]. The antithesis to the concept of scale borrowing is the notion of agglomeration shadow, initially employed to elucidate enterprise development. It denotes that the progress of the vicinity surrounding enterprise clusters is constrained by competitive forces, rendering enterprises situated in the "shadow area" unprofitable [23]. Meijers et al. redefined these two concepts, scale borrowing and agglomeration shadow, within the context of urban networks. Scale borrowing refers to the positive impact of networks on urban economies, wherein small cities promote their own development by leveraging the service functions provided by larger cities. And agglomeration shadow refers to the negative impact of the network on urban economies, wherein small cities are limited in their own development by the siphon effect of large cities after being embedded in the regional network [24]. In the realm of urban network theory, the concepts of "scale borrowing" and "agglomeration shadow" aim to elucidate the mechanism underlying external effects in urban networks by considering cities of varying scales.

### 2.2 Empirical evidence of network externalities

Based on existing studies, it seems that empirical studies on network externalities have focused on the impact of urban networks on urban development. Mechanisms affecting urban development are an important area of research in disciplines such as economic geography, and the spatial agglomeration of economic activities has been recognized as one of the prerequisites affecting urban development [25]. Agglomeration economy generates external effects through the spatial agglomeration of urban elements in the same region. However, such external effects are often restricted by geographical space. Cities can embed themselves into a wider regional network system, such as megalopolis [26], mega-regions [27, 28], city-regions [29], global city-regions [30], the proposed system facilitates the transcending of production activities beyond city boundaries, enabling their operation across interconnected networks spanning multiple cities. As a result, it amplifies the spatial spillover effects associated with agglomeration economies and fosters collaborative development and interaction among cities in the region [31].

In the past decade or so, an increasing number of cities have consciously and actively integrated themselves into urban networks of various levels and scales to share the benefits brought about by such networks. This has sparked a series of studies and concerns regarding the impact of urban networks on regional urban development. Burger, Meijers et al. [20] have identified variations in the impact of agglomeration economies and urban networks on cities at different levels; Thissen et al. [32] argue that a development strategy based on regional networks is more conducive to promoting regional growth compared to one solely focused on location, thus highlighting the stronger role of network connections in driving economic expansion; Liu et al. [33] demonstrate that both agglomeration economies and urban networks contribute to fostering high-quality development in the YRD.

In terms of measuring network externalities, current empirical studies predominantly employ global regression models to estimate urban network externalities by analyzing the spatial spillover effects of factors influencing urban development. For instance, Boix et al. [18], based on the perspective of knowledge innovation, employed the employment growth rate as the dependent variable and considered indicators such as export company scale, labor scale, and transportation cost as independent variables for scale agglomeration. Additionally, they used diversity and specialization level of export companies as independent variables for network connection. By employing a spatial error model, they found that urban employment growth in Catalonia was influenced by both scale agglomeration and network connection and the external effect of agglomeration was more pronounced than that of network connection; Burger and Meijers et al. [20] employed the beta regression model to investigate network externalities, incorporating political, scientific, corporate, cultural, sports, and other comprehensive factors for constructing urban function indicators that represent the level of urban development. Population size and per-capita GDP are utilized as independent variables to signify scale agglomeration, while urban accessibility was used to represent network connection. The study reveals that agglomeration externalities hold greater significance than network externalities; furthermore, compared to small cities, large cities are more likely to benefit from national networks. Sun et al. [34] adopted the spatial Durbin model, taking the growth value of the secondary and tertiary industries as the dependent variable, taking fixed investment, employment scale and other indicators as the independent variables of scale agglomeration, and taking the cumulative total investment between enterprises as the independent variable of network connection. The empirical test indicated that network connection can significantly promote the regional economic growth of China’s major urban agglomerations. However, all these studies were conducted under the assumption of disregarding regional disparities and failed to fully account for the influence of disparate resources and developmental levels within urban areas on the functionality of urban networks.

### 2.3 Research summary

Urban network externality, as a research paradigm elucidating the impact of urban networks on urban development, has garnered significant attention. Previously, spatial agglomeration of economic activities was considered a prerequisite for urban development, and regional networking can expand the scope of spatial spillover effects from agglomeration economies to some extent, subsequently exerting positive or negative influences on the economic development of surrounding areas. These effects vary depending on the level and scale of urban development. Building upon this foundation, the current research on the external effects of urban networks has established a research paradigm that employs regression models to quantify these effects through regression coefficients. In these models, economic efficiency is the dependent variable while various network connection data serve as independent variables. Additionally, production factor indicators such as population size are incorporated as the scale agglomeration variable. However, there are still two issues in this type of research: First, the predominant approach in existing research primarily relies on global regression models, which overlook the differences in network externalities between cities within the region. Second, due to diverse perspectives on urban network research, many scholars tend to select economic factors as dependent variables while including non-economic factors such as traffic as independent variables, resulting in inconsistent variable types.

This paper introduces the MGWR model with the objective of enhancing the measurement of network externalities from a regional perspective down to individual cities within that region, thereby facilitating a more precise comparison of the impact of regional networks on urban development across all levels.

## 3 Methods and data

### 3.1 Research object

**Fig 1:**
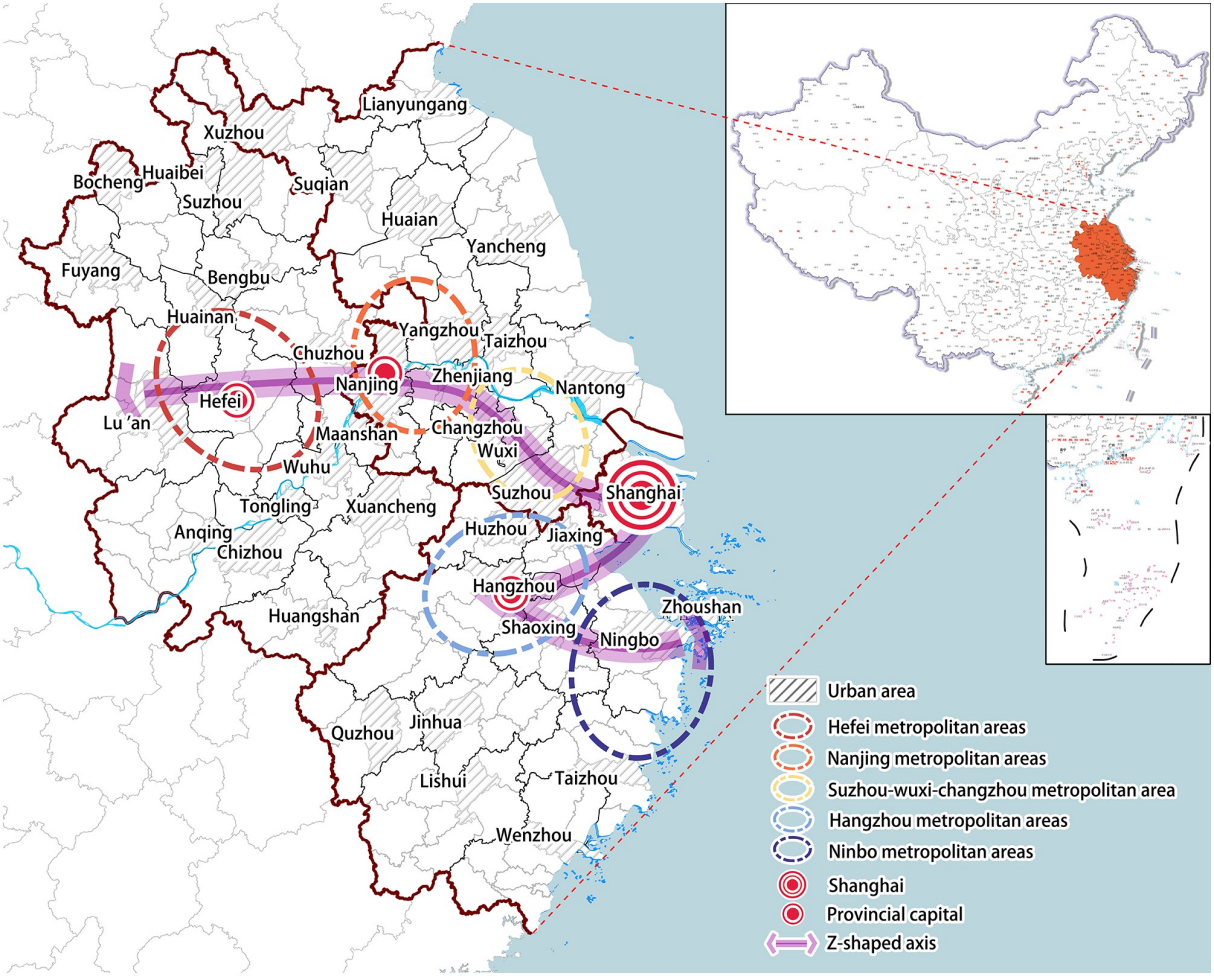
Study area. (Source: created by the author based on the map from Natural Earth (public domain): http://www.naturalearthdata.com/).

With a history spanning over 30 years since the implementation of the regional integration strategy, the YRD stands as one of China’s most dynamic, open, and innovative economic regions. It represents the highest form and direction of China’s regional development, while playing a pivotal role in radiating and propelling the nation’s economic and social progress. The YRD region encompasses three provinces and one municipality directly under the central government, namely Anhui Province, Jiangsu Province, Zhejiang Province and Shanghai Municipality. In China, provinces govern prefecture-level cities, which are comprised of multiple "cities." These cities are classified into "district""county" and "county-level city" based on their size and level of urbanization, all falling within the same administrative hierarchy. The research focus of this paper is on "relatively independent cities" that encompass both "county" and "county-level city". However, the core components of the urban main body and the center of regional economic development are represented by "districts". These districts have contiguous land areas; thus, they are merged as a research unit. Consequently, the YRD region is partitioned into 214 research units based on districts and counties (Fig 1). These study units solely vary in terms of their size and are collectively denoted as cities in subsequent research.

The essence of urban networks lies in the circulation of funds that establish economic connections among different cities [8]. As key market players, enterprises facilitate regional capital flow through cross-regional investments, thereby fostering interconnectivity between cities and driving the development of urban networks. Capital is one of the fundamental drivers of economic growth, and enterprise investment serves as a crucial mechanism for capital control. The efficient flow of capital across cities can effectively allocate scarce factor resources, enhance the overall productivity of cities, and facilitate high-quality urban development. Therefore, it is viable to explore the impact of urban networks on urban development from the perspective of enterprise investment. The intercity enterprise investment data utilized in this paper encompasses all enterprises registered with the State Administration for Industry and Commerce. This paper extracts the investment linkage data between cities from the registered capital information, which is a kind of stock data representing the investment equity relationship. As long as the equity remains intact, the investment will retain its effectiveness. When invested enterprises generate profits, dividends will be distributed proportionally to corresponding investing enterprises. The external effects of urban networks pertain to the advantages that cities derive from their connections [16]. This paper further quantifies these effects as the benefits relative to the scale of a city’s own network connections, i.e., each 1% increase in a city’s network connections can promote or inhibit the development of the city by how many percentages. This approach avoids being influenced by the size of network connections when assessing the strength of network external effects, and facilitates comparisons among cities at different levels.

### 3.2 Benchmark modeling and research data

#### 3.2.1 Variable selection

(1) Dependent variable
  The GDP serves as a direct reflection of the economic magnitude of a city, and economic magnitude stands as a pivotal indicator of urban development. Therefore, this paper utilizes the GDP to represent the level of urban development.
(2) Independent variable
  In the context of globalization, an increasing number of studies have recognized urban networks as a crucial catalyst for promoting urban development [35–37]. This paper employs the economic linkage method introduced by Taylor [8] to represent urban networks and considers cross-city enterprise investment as an independent variable influencing urban development.
(3) Control variable
  According to the traditional theory of endogenous growth [38, 39], urban development is influenced by production factors such as technical knowledge and human capital inherent to the city itself. These factors aggregate within urban space, thereby reducing inter-regional communication costs and serving as a potent driving force for urban development [40]. Therefore, scale agglomeration is an important variable affecting urban development. Drawing upon research methodologies employed by scholars like Boix [18], Meijers [20] and others, this paper assigns weights to certain variables representing production factors to characterize scale agglomeration.

In addition, in the new endogenous growth theory, innovation is also considered as a pivotal driver of economic growth [39, 41]. Innovation has multiple different indicators, among which patents are one of the most commonly used indicators [42, 43]. Therefore, the number of patent authorizations is employed in this paper to serve as an additional control variable for characterizing the level of urban innovation.

#### 3.2.2 Baseline model

The influencing factors of urban development level are measured in this paper by constructing a regression model based on the Cobb-Douglas production function. The prototype formula is as follows:

$$Y=AL^{\alpha }K^{\beta }$$ (1)

In the formula: *Y* denotes gross industrial output value. *A* denotes the comprehensive technology level, *L* denotes the input labor, and *K* denotes the input capital, the *α* and *β* denote the share or elasticity coefficient of labor output and capital output, respectively. Following a logarithmic transformation, the equation is transformed into a multiple linear regression model expressed by the following formula.


lnY=lnA+αlnL+βlnK (2)

According to variable selection, the urban development level replaces the gross industrial output value, while scale agglomeration, network connection, and innovation level replace input labor and input capital.

The level of urban development is quantified by GDP, while the scale of agglomeration is quantified by factors such as labor force scale, fixed asset investment, total social retail consumption, and construction land scale, which represent the input scale of production factors. The network connection is represented by the point degree centrality, which quantifies the importance of nodes in the urban network. It is calculated as the sum of cross-city outward investment and absorbed investment of enterprises aggregated by cities. The innovation level is quantified by the number of patent authorizations. The above formula can be transformed as follows:

$$lnD=\beta_{0}+\beta_{1}\;ln\;SA+\beta_{2}\;ln\;NC+\beta_{3}\;ln\;IL+\varepsilon$$ (3)

In the formula: *D* denotes the level of urban development. *SA* denotes scale agglomeration, *NC* denotes network connection, *IL* denotes the level of innovation. *β*_1_, *β*_2_ and *β*_3_ denote the external effects of agglomeration, the external effects of network and the effect of innovation level. In order to eliminate the influence of the scale on the results, the variables are standardized by Z-value respectively, i.e., all the variables are scaled to the values with mean 0 and standard deviation 1.

The four independent variables characterizing scale agglomeration are normalized by MaxAbs and summed according to the same weights and then logarithmically processed, and the variable calculations are detailed in Eq (4).


$$\begin{array}{l} lnSA\\ =ln[MaxAbs\left(Pop\right)+MaxAbs\left(Fix\right)+MaxAbs\left(Sales\right)\\ +MaxAbs\left(Area\right)] \end{array}$$ (4)

In the formula: *SA* denotes scale agglomeration; *MaxAbs*(*Pop*), *MaxAbs*(*Fix*), *MaxAbs*(*Sales*) and *MaxAbs*(*Area*) denote the normalized values of the number of labor force, the scale of fixed asset investment, the total retail sales of consumer goods, and the area of construction land, respectively.

The two independent variables representing network connectivity are logarithmically transformed after summation, and the variable calculations are detailed in Eq (5).


$$lnNC=ln\left(I_{out}+I_{in}\right)$$ (5)

In the formula: *NC* denotes network connection; *I*_*out*_ denotes the size of cross-city outward investment by firms aggregated by city; *I*_*in*_ denotes the size of cross-city investment absorption by enterprises aggregated by city.

The independent variable representing the level of innovation contains "0" values. To ensure meaningfulness and maintain data structure integrity, a logarithmic transformation is applied after adding 1 to all the data, and the calculation of the variables is detailed in Eq (6).


lnIL=ln(Patent+1) (6)

In the formula: *IL* denotes the level of innovation; *Patent* denotes the number of domestic patents granted.

#### 3.2.3 Research data

The data is categorized into two groups: statistical data and enterprise data. Statistical data and sources, including GDP, labor force, and fixed asset investment scale, are obtained from the 2021 Statistical Yearbook of Chinese Provinces (http://www.tongjinianjian.com/). Total retail sales of social consumer goods are sourced from the 2020 Statistical Communique of National Economic and Social Development of Districts and Counties (http://district.ce.cn/), while the number of patent authorizations in cities is derived from the National Intellectual Property Administration (https://www.cnipa.gov.cn/).

Enterprise data and sources: The data on enterprise investment scale is based on the industrial and commercial registration information of enterprises obtained from Tianyancha (https://www.tianyancha.com/). It includes the effective investment data of enterprises that are still operational in 214 cities within the YRD as of December 31, 2020, amounting to a total of 1.3033 million records and 54.8 trillion yuan. This dataset encompasses all enterprises registered with the State Administration for Industry and Commerce, providing a comprehensive sample without any sampling bias. These investment data include investment between cities within the YRD, investment by cities within the YRD to cities outside the YRD, and investment by cities outside the YRD to cities within the YRD.

**Fig 2:**
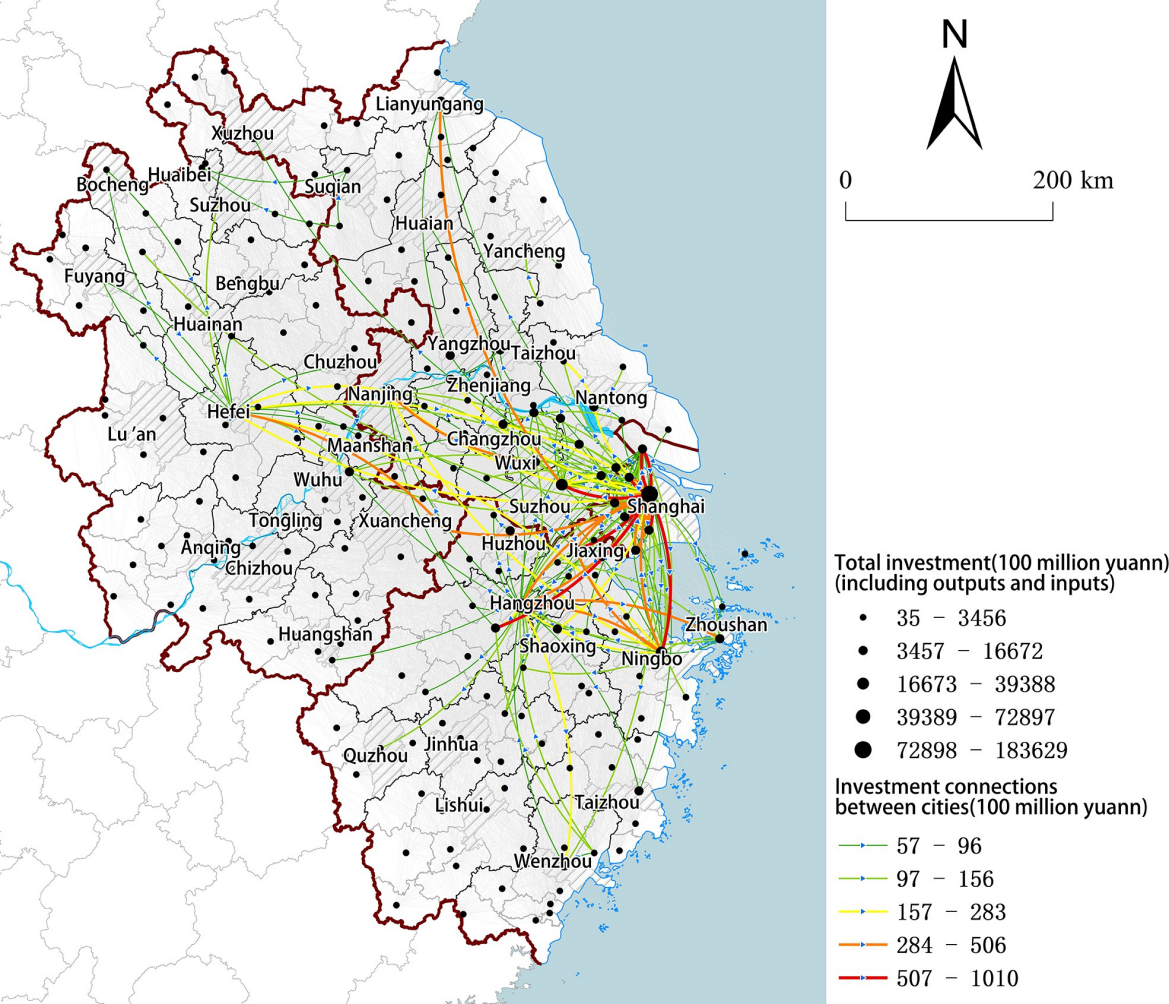
Urban networks of the YRD region. (Source: created by the author based on the map from Natural Earth (public domain): http://www.naturalearthdata.com/).

According to the research unit of this paper, the aforementioned data have been organized into the fundamental dataset (Table 1), while the urban networks are constructed by aggregating cross-city investments (Fig 2).

**Table 1:** Basic data.

City	Gross domestic product (100 million yuan)	Number of labor force (ten thousand people)	Construction land area (km)	Investment in fixed assets (100 million yuan)	Total retail sales of social consumer goods (100 million yuan)	Number of authorized invention patents (item)	Outward Investment (100 million yuan)	Absorbed Investment (100 million yuan)
Anqing urban area	548.16	80.45	89.03	2575.57	416.67	44.00	681.38	779.19
Huaining	312.27	49.67	63.91	324.50	117.69	53.00	67.95	98.46
Qianshan	216.71	44.12	33.62	217.45	110.97	16.00	53.36	64.33
Susong	243.15	61.26	72.67	279.07	99.02	11.00	57.67	75.68

#### 3.3 Measures of externalities

The current research models on network externalities primarily focus on measuring the overall external effects within a region. To evaluate the benefits of individual cities integrated into the urban network within that region, it becomes imperative to employ a localized regression model. Therefore, this paper employs the MGWR model for empirical analysis. The formulated model is presented as follows:

$$y_{i}=\overset{m}{\underset{j=1}{\sum }}\beta_{bwj}\left(u_{i},v_{i}\right)x_{ij}+\varepsilon_{i}c$$ (7)

In the formula: *y*_*i*_ denotes the dependent variable of the *i* th city; (*u*_*i*_, *v*_*i*_) denotes the coordinates of the coordinates of the *i* th district or county, using the location of the seat of government to locate it; *x*_*ij*_ denotes the *i* th independent variable of the *j* th district and county; *bwj* denotes the bandwidth of the *j* th variable, and *β*_*bwj*_ denotes the regression coefficient of the *j* th variable; *ε*_*i*_ denotes the error term for the *i* th district or county; *m* denotes the number of variables in the regression model.

The underlying assumption for employing this methodology is the presence of spatial correlation in the dependent variable. This paper conducted the global spatial autocorrelation analysis on the gross domestic product (GDP) data from 214 cities, revealing that Moran’s I index yielded a value of 0.552 (significantly passing the 1% threshold), the necessary conditions have been met.

The collection and analysis method complied with the terms and conditions for the source of the data.

## 4 Results

### 4.1 Model analysis

#### 4.1.1 Covariance tests and variable screening

Prior to conducting the MGWR model test, it is imperative to perform a collinearity assessment on the variables using SPSS software based on the multiple linear regression model. The variance inflation factor (VIF) test results for each parameter are presented in Table 2. It is evident from the table that the VIF values of the independent variables range between 2.571 and 4.034, all below the threshold of 10, indicating an absence of multicollinearity among these variables.

**Table 2:** Collinearity test.

Variable Type	Characterization variable	Variable abbreviation	Collinearity statistics VIF
Dependent variable	Urban development level	D	—
Independent variable	Network connection	NC	3.617
Control variable	Scale agglomeration	SA	4.034
	Innovation level	IL	2.571

#### 4.1.2 Comparison of analysis results

In order to examine the external impacts of resource factor agglomeration and network connections in each city, this paper employs the MGWR model for further analysis. The output results are compared with the OLS model (Table 3). In the OLS model, all coefficients of both the independent variables and control variables passed the significance test at a 1% level. The goodness of fit measures R^2^, AICc, and the sum of squares for the MGWR model exhibited improvements when compared to those of the OLS model. Consequently, it can be concluded that the results obtained from the MGWR model are superior. Furthermore, the local condition numbers of each city are all below 30, indicating the absence of local collinearity in the data.

**Table 3:** Regression model indicators.

Norm	MGWR	OLS
Goodness-of-fit R^2^	0.927	0.895
AICc	77.912	133.145
Sum of squares of the residuals	13.845	22.247
Standardized residual sum of squares	0.270	-

### 4.2 Interpretation of results

#### 4.2.1 Capital agglomeration remains a key driver of urban development

According to the final fitting results of the model, the standard regression coefficients for scale agglomeration and innovation level in all 214 cities passed the 10% significance test. However, only in 152 cities did the network connection standard regression coefficients pass this same significance test. The average standard regression coefficient for scale agglomeration is 0.701 (Table 4), which significantly higher the coefficients of other variables, and all sample coefficients are positive. These findings suggest that scale agglomeration remains the primary driving force behind urban development: scale agglomeration can facilitate the development of all cities; however, only 71% of cities can utilize network connections to promote urban development. The findings of this paper are consistent with the research conclusions of Boix et al. [18] and Wetwitoo et al. [44], that the external impact of scale agglomeration surpasses that of network in influencing urban development.

**Table 4:** Standardization coefficient statistics.

Independent variable	Bandwidths	Average value	Minimum value	Maximum values	Number of cities with P≤0.1	Percentage of cities with P≤0.1 (%)
Scale agglomeration	212	0.701	0.690	0.711	214	100
Network connection	50	0.131	0.101	0.286	152	71
Innovation level	212	0.090	0.076	0.104	214	100

Note: The data in the table are statistics for samples that passed the test of significance.

Regional networking can contribute to urban development to a certain extent. By establishing network connections, cities can expand the spatial scope of agglomeration economies, thereby acquiring additional production factors such as technology and talent, leading to further breakthroughs in development. However, based on the findings of this paper, the external effects of networking are not as significant as those resulting from internal scale agglomerations within cities: for every 1% increase in investment in production factors, GDP can be increased by 0.690%-0.711%; whereas for every 1% increase in enterprise investment, GDP only rises by 0.101%-0.286%. Considering the standard coefficient ratio between network connections and scale agglomerations when promoting urban development levels, it is estimated that network connections play a role equivalent to approximately one-seventh to two-fifths of capital agglomeration.

#### 4.2.2 The urban network effect demonstrates characteristics of spatial heterogeneity

According to the results of the MGWR model, different variables have different bandwidths (Table 4). The size of the model bandwidth reflects the strength of spatial heterogeneity of influencing factors. When the bandwidth value is close to the total number of samples, it indicates that the spatial heterogeneity of influencing factors is weak.

The bandwidth of the two variables, scale agglomeration and innovation level, is 212 samples, which is close to the full sample. The standard regression coefficients for these variables range from 0.690 to 0.711 and from 0.076 to 0.104 respectively, with coefficients of variation of 0.010 and 0.104 respectively, indicating minimal differences in standardized coefficients and no spatial heterogeneity present. In contrast, the bandwidth for network connection is limited to only 50 samples. Among the subset of cities (152) that pass the significance test, the standard regression coefficients vary significantly from a minimum value of 0.101 to a maximum value of 0.286, resulting in a coefficient of variation equaling 0.280. The substantial difference in standardized coefficients suggests spatial heterogeneity exists in terms of external effects derived by these cities through their integration into the urban network.

**Fig 3:**
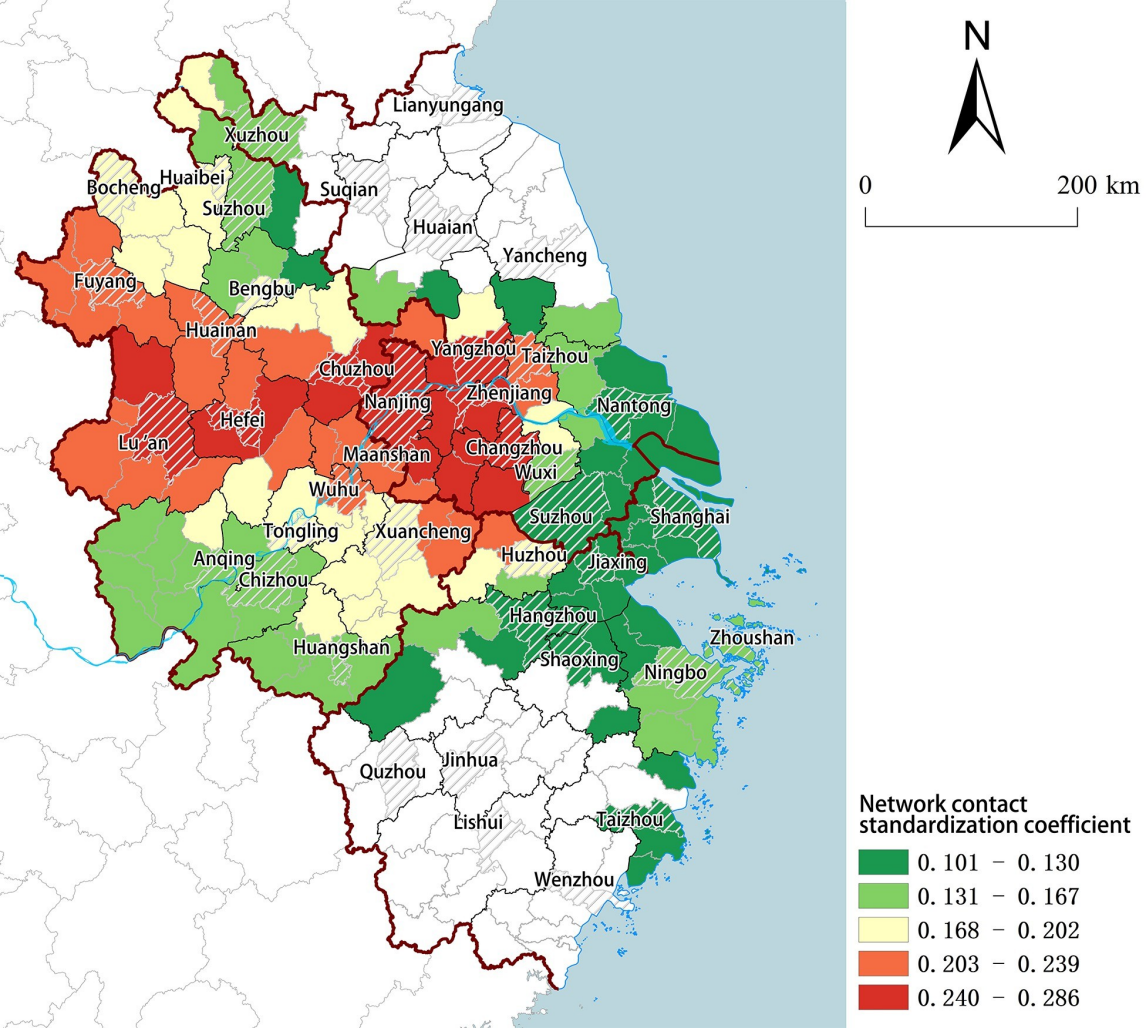
Spatial distribution of network externalities. (Source: created by the author based on the map from Natural Earth (public domain): http://www.naturalearthdata.com/).

The investment network significantly and positively impacts cities concentrated in the central area of the YRD (Fig 3), this scope encompasses the entire province of Anhui (excluding Si County), southern Jiangsu, northern Zhejiang, Shanghai, and the coastal zone of eastern Zhejiang. Moreover, these cities are predominantly clustered within the core areas of the five major metropolitan regions outlined in the "Yangtze River Delta Urban Agglomeration Development Plan".

By comparing Fig 3 with the scope of the metropolitan area depicted in (Fig 1), it becomes apparent that the five metropolitan areas experience varying benefits from their integration into the urban network due to disparities in developmental factors. The Hefei Metropolitan Area and Nanjing Metropolitan Area demonstrate the strongest network external effect, as regional networking has facilitated substantial developmental advantages and access to external resources for these areas. Over a period exceeding ten years, both the Hefei Metropolitan Area and Nanjing Metropolitan Area have consistently observed year-on-year increases in economic aggregate, establishing themselves as the most economically advanced, dynamic, comprehensive, and open regions within their respective provinces, they serve as pivotal growth poles for provincial development.

According to the average level of external effects in the network across different provinces (Table 5), the strength of external effects varies from high to low for Anhui Province, Jiangsu Province, Zhejiang Province, and Shanghai. By conducting a significance test on the proportion of cities, it is observed that Shanghai and Anhui Province have the highest number of cities passing the test, followed by Jiangsu Province, while Zhejiang Province has the fewest. In terms of both intensity and prevalence of external effects, Anhui Province outperforms other provinces. These findings indicate that the integration into the YRD city network has significantly propelled urban development in Anhui Province, aligning with its robust recent GDP growth.

**Table 5:** External effects of network connections across provinces.

Province	Average value	Minimum value	Maximum values	Number of cities with P≤0.1	Proportion of cities with P≤0.1 in the province (%)
Anhui	0.1934	0.1183	0.2551	78	98.73%
Jiangsu	0.1901	0.1081	0.2857	37	61.67%
Zhejiang	0.1335	0.1009	0.2071	30	44.12%
Shanghai	0.1092	0.1063	0.1119	7	100.00%

Specifically (Table 6), the majority of cities exhibiting strong network externalities within each province do not conform to the traditional notion of high-level cities, such as Jintan, Liyang, Danyang and Jurong in Jiangsu Province; Feidong, Quanjiao and Lai’an in Anhui Province; Changxing, Anji, Deqing and Xiangshan in Zhejiang Province, and so on. These cities, although not considered high-level, exhibit a stronger network external effect compared to high-level cities. When considering the Z-shaped axis in the Development Planning of the YRD City Group (i.e. Shanghai-Nanjing-Hefei-Hangzhou-Ningbo Development Belt) (Fig 1), it becomes evident that cities with the most pronounced network external effects are aligned along this axis and distributed in a bead-like pattern, relying on the Z-shaped axis. The network external effects within Anhui Province display significant variations, with areas of high network external effects concentrated along the Z-shaped axis that traverses through Hefei, Liu’an, and Chuzhou urban areas. Similarly, Jiangsu Province’s cities demonstrate similar characteristics, where Nanjing and Changzhou serve as core urban areas from which these effects gradually diminish outwardly. Both Shanghai and Zhejiang exhibit weak network external effects with minimal differences among their respective cities; however, regions with such effects remain concentrated along the Z-shaped axis.

**Table 6:** Top five districts and counties in terms of external effects of network in each province.

Province/Municipality	Prefecture-level city	County/District/County-level city	Standardization coefficient (network external effect)	Province/Municipality	Prefecture-level city	County/District/County-level city	Standardization coefficient (network external effect)
Jiangsu	Changzhou	Jintan	0.286	Zhejiang	Huzhou	Changxing	0.207
	Zhenjiang	Danyang	0.284		Huzhou	Anji	0.196
	Zhenjiang	Zhenjiang urban area	0.281		Huzhou	Huzhou urban area	0.171
	Zhenjiang	Jurong	0.280		Ningbo	Xiangshan	0.164
	Changzhou	Liyang	0.270		Huzhou	Deqing	0.162
Anhui	Lu’an	Lu’an urban area	0.255	Shanghai	Shanghai	Jinshan	0.112
	Chuzhou	Chuzhou urban area	0.253			Fengxian	0.111
	Hefei city	Feidong	0.251			Jiading	0.109
	Chuzhou city	Quanjiao	0.249			Shanghai urban area	0.109
	Chuzhou	Laian	0.249			chongming	0.109

#### 4.2.3 Major cities play a radiation-driven role

**Fig 4:**
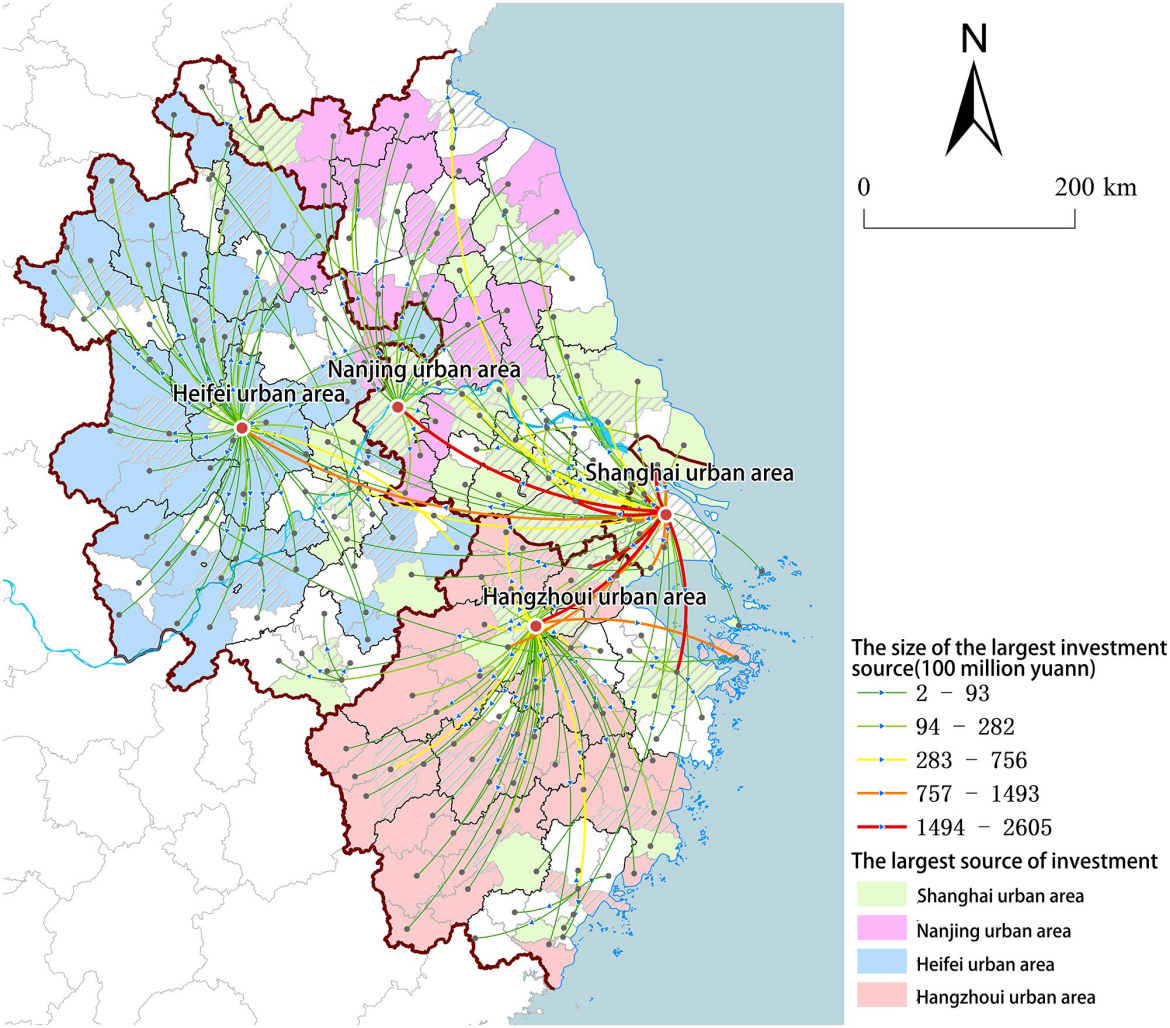
The largest source of investment. (Source: created by the author based on the map from Natural Earth (public domain): http://www.naturalearthdata.com/).

The city network of the first source of investment is constructed by selecting the largest source of investment as the primary one among all sources in each city (Fig 4). The urban regions of Shanghai, Hangzhou, Nanjing, and Hefei are the key nodes in the network, accounting for more than 76% of total investment in the region. Specifically, Shanghai’s urban region serves as the primary source of enterprise investment for the other three major cities. In general, enterprise investment radiates around core nodes within various provincial spaces and exhibits structural characteristics such as "multi-center""multi-level" and "center agglomeration". Further examination of the primary investment link between cities reveals that southern Jiangsu Province, owing to its proximity to Shanghai, has been significantly influenced by the spill-over effect emanating from Shanghai. The predominant source of investment for most cities in this region is derived from the urban area of Shanghai, while cities in Zhejiang and Anhui provinces continue to rely on their respective provincial capitals as their main source of investment.

In order to assess the leading role of high-level cities in regional radiation, this paper conducted a model reanalysis by excluding data pertaining to the aforementioned four cities. The results indicate that the standardized regression coefficients representing network connections are not statistically significant. This implies that high-level cities effectively promote regional development through investment and achieve a desirable radiation effect, aligning with the objective of regional integration in the YRD.

### 4.3 Robustness test

**Fig 5:**
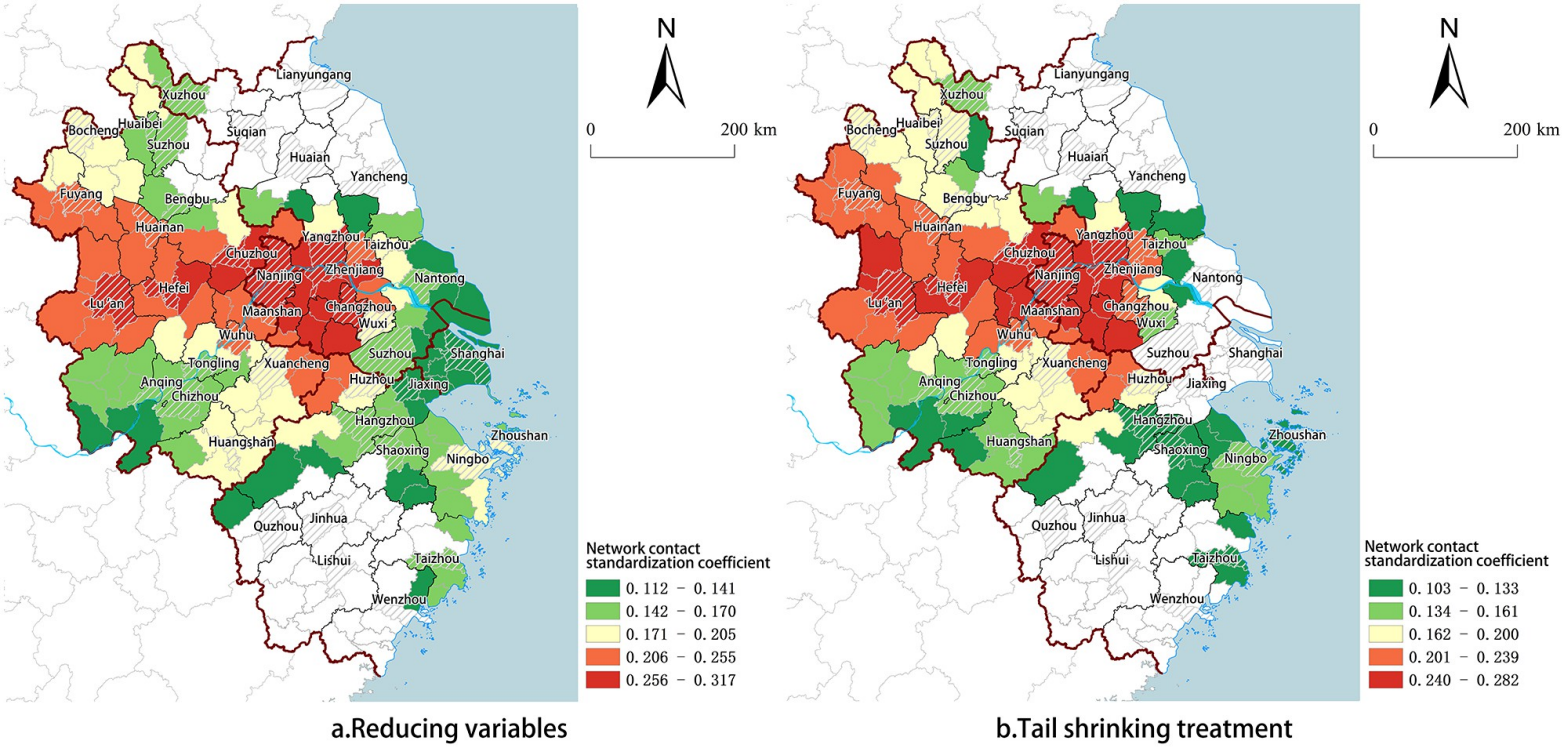
Robustness test results. (Source: created by the author based on the map from Natural Earth (public domain): http://www.naturalearthdata.com/).

(1) Tail shrinking treatment
  With the sample size remaining unchanged, extreme value processing is conducted at the 1% and 99% quantiles, wherein values below the 1st percentile are substituted with values at the 1st percentile, while values exceeding the 99th percentile are replaced with values at the 99th percentile. The range of variation among the indicators in Table 7 is relatively small, and all standard regression coefficients for scale agglomeration pass a significance test at the 10% level. While the number of cities with network connection coefficients passing the significance test has decreased from 152 to 129, overall spatial distribution characteristics remain essentially unchanged (Fig 5).

**Table 7:** Indexes of robustness test.

Norm	Model 1 (Original model)	Model 2 (Tail shrinking treatment)	Model 3 (Reducing variables)
Goodness-of-fit R^2^	0.927	0.925	0.926
AICc	77.912	83.495	80.32
Sum of squares of the residuals	13.845	14.211	14.128
Average value of agglomeration externalities	0.701	0.694	0.754
Average value of network externalities	0.131	0.128	0.144

(1) Reducing variables
  Re-run the model by excluding the independent variable representing the urban Innovation level. According to the results (Table 7), the agglomeration scale coefficients of all cities have successfully passed the significance test, and network connection coefficients of 153 cities have also demonstrated significant results. Overall, both the spatial distribution characteristics and coefficient magnitudes exhibit no substantial deviations from those in the original model (Fig 5).

The results of the above robustness tests indicate that the results of the study are reliable.

## 5 Discussion

The economic aggregate of the YRD region has consistently accounted for 1/4 of the national total, with a remarkable increase from 22.1 trillion yuan in 2018 to 29 trillion yuan in 2022. It serves as a robust driving force underpinning China’s economy, exhibiting a synergistic effect where "1+1+1+1>4." In order to investigate the correlation between rapid urban development and the urban network in the YRD recently, this paper examines the impact of regional networks on the urban development of the YRD from the perspectives of agglomeration externalities, network externalities, and the differences in spatial performance of the two effects.

### 5.1 The importance of networking

The research findings indicate that while regional networking has a positive impact on urban development to some extent, scale agglomeration remains the primary driving force behind urban development. This implies that urban development in the YRD region predominantly relies on local strengths, such as long-term capital accumulation, technological advancements, and availability of talented resources. These factors establish the foundation and essential prerequisites for urban development, aligning with the significance of agglomeration emphasized by traditional urban systems [20, 45] and supporting endogenous growth theory [38, 39].

However, in the current era of networking, the influence of network connections is increasingly significant [20]. To enhance cities’ competitiveness in global competition, it is imperative to enhance international connectivity through the integration of networks of various scales [46]. Currently, most regions in the YRD have successfully attained positive network externalities, signifying that the implementation of regional integration policies has yielded significant outcomes. The emergence of urban networks’ external effects is predicated on the internalization of external resources, including labor, capital, technology, and management. In other words, cities leverage the network to convert external resources into local production factors, effectively driving the transformation and advancement of local manufacturing and high-tech industries while enhancing overall factor productivity. Intercity investment by enterprises has emerged as a crucial strategy for accessing external resources, transcending the constraints of single city development. However, effectively internalizing such investments and harnessing them to meet local developmental needs necessitates proactive allocation and integration efforts by city governments at all levels, thereby unleashing their inherent agency.

### 5.2 Spatial heterogeneity

Considering the varying levels of development and inherent conditions among cities, as well as their capacity to harness resource endowments and external investments, the impact of each 1% increase in factor input on urban development levels exhibits heterogeneity. The spatial variations in the external effects of agglomeration within the YRD are not found to be statistically significant, which contrasts with previous scholarly findings suggesting that larger cities exhibit stronger agglomeration externalities [47]. This discrepancy can be attributed to differences in measurement standards, as this paper employs relative values to assess the impact of scale agglomeration on urban development. In terms of prefecture-level cities in Hangzhou, both the urban area of Hangzhou and Chun’an County exhibit a standardized coefficient of agglomeration approximately equal to 0.71. This implies that for every 1% increase in input of production factors, the GDP will experience a corresponding growth rate of 0.71%. Despite their apparent similarity, it is important to note that due to its larger GDP base, even a mere increment of 0.71% in the urban area of Hangzhou translates into an equivalent value of RMB 950 million (100 million yuan); whereas for Chun’an County, this amounts to only RMB 170 million (17 million yuan). Therefore, from this perspective, the results still adhere to the general principle that "the larger the size of a city, the stronger its agglomeration external effect." However, when considering the YRD region with significant disparities in development levels among cities themselves, relying solely on scale agglomeration is evidently insufficient for achieving balanced development across different tiers of cities.

What are the distinguishing characteristics of urban network externalities? The findings of this paper unequivocally validate the positive impact of the embedded urban network on the development of most cities, showcasing distinct characteristics of spatial heterogeneity. However, irrespective of the metropolitan area, provincial level, or the smallest research unit, low-level cities demonstrate greater efficiency in enhancing their urban development level through urban networks compared to high-level cities. This finding aligns with the research conclusions drawn by Camagni et al.[4]. In terms of the internalization and absorption of investment, low-level cities with robust network externalities are situated near high-level cities such as Hefei, Nanjing, and Hangzhou. These cities can effectively leverage the production factors and larger and more extensive capital markets available in high-level cities. By integrating their internal advantageous industrial resources, they can undertake high-end industries from these advanced urban centers, thereby contributing to the enhancement of urban development quality and efficiency. Previous studies have demonstrated that strengthening industrial functional complementarity and synergy typically amplifies the positive impact of networks [48]. Taking Changxing as an example, it serves as a prominent industrial county and a demonstration zone for industrial transformation and upgrading in Zhejiang Province. The study reveals the county’s robust external effects, with each 1% increase in investment links contributing to a 0.207% rise in its GDP. Considering the practical circumstances, Changxing has attracted significant investments of 165.4 billion yuan from enterprises such as Suzhou Aikang Technology, Hangzhou Geely Group, and Shanghai Changfeng Group. By actively promoting the development of new energy industry clusters to replace the previously high-polluting battery industry, it currently plays a pivotal role within the new energy industry chain in the YRD. In recent years, Changxing has hitherto accomplished rapid and robust urban economic development by effectively harnessing resources acquired through the investment network, thereby consistently securing its position as the leading industry in Huzhou. In contrast, despite Lianyungang in Jiangsu province attracting a total investment of 301.5 billion yuan (exceeding local outbound investment), it surpasses the amount of investment attracted by Changxing County by 1.8 times. However, the peripheral location of the region within the YRD and limited support from neighboring metropolises pose challenges for its access to the capital market of prominent cities and internalization of investments. Additionally, Lianyungang’s current industry is predominantly composed of traditional sectors such as heavy chemicals, which are challenging to integrate with modern high-end leading industries such as electronic information, biomedicine, and new energy in the YRD region. Consequently, the absence of robust connections between city and investment networks impedes the efficient internalization and assimilation of external capital.

### 5.3 Suggestions to improve network externalities

Firstly, the investment network transcends the constraints of individual city development and serves as a catalyst for urban progress. Hence, during the pivotal phase of the "14th Five-Year Plan" advancement, it is imperative to steadfastly execute the strategy of regional coordinated development. Currently, China’s large-scale regional development strategies, such as the integrated development of the YRD and the economic belt development along the Yangtze River, have yielded certain outcomes, gradually establishing a nationwide pattern of multi-level, multi-form, and comprehensive regional coordinated development. However, there is room for improvement in terms of precision and targeted implementation of regional policies. In future regional development, greater attention should be paid to the demonstration effect of small-scale, cross-regional, and relatively accurate regional cooperation. Drawing inspiration from the ’adjacent development model of ’a big tree offers good shade’ in the surrounding areas with Shanghai as the center,’ promoting the multi-dimensional coupling of administrative boundaries, geographical boundaries, economic boundaries and socio-cultural boundaries based on small-scale space, and explore new driving forces for regional integration in the coordination of material civilization and spiritual civilization, and the harmonious coexistence of human and nature.

Secondly, the impact of investment networks varies across regions, necessitating the optimization of investment strategies based on the unique developmental conditions in each specific region. Large cities such as Shanghai and Suzhou should prioritize the development of their central city status, enhance their influential reach, relocate low-tech production functions to peripheral regions, and concentrate on high-value segments in the industrial value chain including headquarters economy, core technologies, innovative applications, and independent research and development. These efforts aim to facilitate the progress of surrounding regions; The northern Jiangsu region, the southern and northern Anhui regions, as well as other small and medium-sized cities should proactively integrate into high-level open cities such as Shanghai, Nanjing, Hangzhou, Hefei, and Suzhou. They should leverage local comparative advantages to replicate and promote new experiences in opening up while continuously enhancing the business environment. Simultaneously, they should actively participate in regional industrial chains and supply chain systems to share the achievements of advanced openness. This will facilitate the complementary development of urban functions and industrial dislocation while elevating regional specialization levels with the aim of narrowing economic disparities.

Thirdly, external investment represents a valuable opportunity for development, necessitating policymakers’ increased attention towards establishing an enabling market environment to ensure the efficacy of cross-regional investment connections. This will facilitate the flow of production factors such as capital across geographical boundaries through investment networks, aligning them with cities’ comparative advantages and thereby promoting the transformation and upgrading of local traditional industries while enhancing production efficiency. The communication effectiveness among multiple local governments should be enhanced to ensure the formulation of reasonable and orderly cooperation guidance policies in guiding the spatial layout of influencing factors, which align with the local economic development stage. For instance, through the joint establishment of industrial parks and promotion of industrial transfer, as guided by the Implementation Plan for Shanghai-Suzhou-Zhejiang City Pair Cooperation in Assisting Northern Anhui Cities, collaboration and support are extended to less developed areas like northern Anhui cities, injecting new impetus into their opening-up endeavors.

## 6 Conclusions

China is not only the second-largest economy in the world but also a developing country with the strongest comprehensive strength. The development of China holds significant reference value for the future progress of other developing countries. With the transformation of China’s economic structure and development mode, regional networking development has become an important means to enhance cities’ external competitiveness and promote urban development. The YRD, as one of the six global metropolitan areas, stands out as China’s most vibrant economic region with the highest population density, greatest openness, and strongest innovation capacity. It epitomizes the pinnacle and trajectory of urban clusters in China while playing a pivotal role in radiating influence and propelling the nation’s economic and social development. Simultaneously, the YRD serves as a paradigmatic exemplification of regional network development in China. Conducting a scientific and precise evaluation of the impact of urban networks on urban development, along with their regional disparities, not only furnishes an empirical case for global research on urban networks within China but also offers valuable insights for other developing nations to foster regional advancement, enhance urban competitiveness and cohesion. This holds significant implications for implementing regional development strategies and informing future decision-making in diverse regions. Based on enterprise investment data, this paper aims to investigate the differential impact of regional networking on the development of the YRD, it incorporates cross-city capital flows as an independent variable to explore the external effects of the network. The main conclusions are as follows:

1. The regional networking plays a significant role in urban development, albeit not as the predominant factor. Every 1% increase in investment links can enhance a city’s development level by approximately 0.101%-0.286%, accounting for around one-seventh to two-fifths of the impact brought about by capital agglomeration. Urban development primarily relies on the agglomeration of resources and factors within the city. However, in order to achieve the coordinated goal of the YRD integration strategy, small and medium-sized cities need to leverage external factors such as network connections to facilitate urban development, given their limited resources.
2. Cities with diverse geographical locations and varying levels of development can derive disparate benefits from network connection. Most low-level cities can achieve traditional industrial upgrading by attracting high-end industries from high-level cities, thereby gaining stronger network externalities than high-level cities. Consequently, embedding urban networks emerged as an effective approach to facilitate the rapid development of low-level cities and foster balanced regional development among cities.
3. Major cities play a leading role in the urban network. The urban areas of Shanghai, Hangzhou, Nanjing, and Hefei serve as the primary sources of investment for cities across various provinces. Leveraging the investments from these major cities enables small and medium-sized cities to overcome resource limitations and leverage networks to foster urban development.

Urban development is a dynamic and intricate process. This paper establishes an urban network using enterprise investment data obtained from a singular temporal cross-section in 2020, aiming to investigate the influence of regional investment networks on urban development. However, it should be acknowledged that this approach overlooks potential time heterogeneity characteristics of network external effects. In future research, incorporating multiple time panel data could enable a comparative study of the evolutionary dynamics of urban network external effects.

In addition, the impact of networks on urban development varies across different scales. High-level cities may exhibit a greater reliance on High-level urban networks (such as the World Urban Network), whereas smaller cities tend to derive more benefits from smaller-scale regional networks [20, 49]. This paper solely focuses on the YRD region, and the network connection only considers outward investment by enterprises registered in China, without presenting the potential benefits that multinational enterprise investment can bring to high-level cities. Further research can delve into whether high-level cities are better positioned to benefit from a wider range of networks.

## Supporting information

S1 Data (XLSX)
